# Effects of different rice straw returning methods in karst paddy fields on soil bacterial community structure and rice yield: a mechanistic analysis based on *16S rRNA* sequencing

**DOI:** 10.3389/fmicb.2025.1710332

**Published:** 2025-12-16

**Authors:** Yueyue Tang, Zhao He, Jiajia Zhou, Hu Wang

**Affiliations:** Guizhou Chuyang Ecological Environmental Protection Technology Co., Ltd., Guiyang, China

**Keywords:** straw return to fields, karst paddy fields, bacterial communities, *16S rRNA* sequencing, rice yield, community assembly

## Abstract

The effective utilization of crop straw can contribute to sustainable agricultural development. However, how different straw return methods regulate soil fertility and rice yield via bacterial communities in karst paddy fields remains elusive. This field study investigated five straw return treatments [deep plowing (PD); rotary tillage with incorporation (RTM); field rapid composting, (FRC); no-till mulching (NT); and bioreactor (BR)] and a blank control CK (no straw return, fertilizer only) on soil physicochemical properties, bacterial community structure, and rice yield, combined with *16S rRNA* sequencing technology. Results indicate the following: (1) all straw incorporation treatments significantly increased soil organic matter (SOM) and nutrient content (*p* < 0.05), NT and BR treatments increased soil organic matter (SOM) by 38.2 and 36.4%, respectively, compared to CK, while total nitrogen increased by 42.1 and 48.4% with NT; (2) although RTM treatment did not achieve the highest SOM accumulation, it yielded the highest rice yield of 30.37 kg/plot (a significant increase of 13.2% compared to CK), revealing that yield is jointly regulated by soil physicochemical properties and bacterial communities; (3) straw return treatments did not significantly affect bacterial α-diversity (intergroup differences in Shannon index and Chao1 index, *p* > 0.05), but significantly influenced β-diversity, symbiotic network structure, and community assembly processes. BR treatment formed a complex and stable microbial network structure, while NT exhibited a highly modular community structure (modularity = 0.66); (4) bacterial community assembly under straw return was dominated by deterministic processes, with homogenous selection accounting for 45 and 42% in NT and BR treatments, respectively, significantly higher than CK (28%, *p* < 0.05); (5) pathwise linear structural equation modeling (PLS-SEM) confirmed that TN (path coefficient 0.97, *p* < 0.001) and bacterial β-diversity (path coefficient 0.83, *p* < 0.001) were the most critical factors influencing rice yield. This study elucidates the mechanisms by which different straw return methods drive soil functions by reshaping bacterial community assembly and interaction networks. It provides theoretical support for optimizing straw return technologies in karst paddy fields, such as applying RTM for “yield-priority” scenarios and NT for “Rapid fertilization” scenarios.

## Highlights

- Straw return methods do not affect α-diversity but influence soil bacterial community structure, network stability, and assembly processes.
- RTM yields the highest under non-optimal soil nutrient conditions, highlighting a microbe-mediated synchronous nutrient release mechanism.
- NT and BR treatments most effectively enhance soil organic matter and total nitrogen, primarily driven by specific microbial groups.
- Deterministic processes dominate microbial community assembly under straw return, with enhanced homogenous selection in NT and BR treatments.
- Total nitrogen and β-diversity are the strongest factors influencing rice yield, underscoring the synergistic interaction between soil nutrients and microorganisms.

## 1 Introduction

Rice (*Oryza sativa*) is one of the world’s essential staple crops. According to statistics, it meets over 21% of the global population’s caloric needs (Fitzgerald et al., 2009). Karst regions are characterized by soluble rock strata (such as limestone and dolomite) as their primary geological formation, with fragmented topography and shallow soil layers as key features (Zhu et al., 2012). Rice paddies serve as the core vehicle for food security in these areas, facing unique ecological constraints. The high bulk density, severe nutrient leaching, low organic matter reserves, and susceptibility to erosion of these soils render the agricultural ecosystem more vulnerable to cultivation impacts (He et al., 2008). Traditional over-cultivation and single-nutrient fertilization further exacerbate soil structure degradation and fertility loss, severely threatening the sustainable development of karst paddy ecosystems (Kang et al., 2024). Straw incorporation into fields serves as an effective measure for resource recycling and ecological conservation. Its role in enhancing soil organic carbon sequestration, regulating nutrient cycling, and reducing environmental risks (Zheng et al., 2022; Zhang et al., 2024) has gained widespread recognition, making it an indispensable technology for sustainable agricultural development in karst regions.

Straw return technology has evolved beyond traditional methods, with diverse approaches now developed and adopted for specific agroecosystems. Its application in karst paddy fields shows a clear developmental trend. Conventional tillage methods, such as deep plowing, can significantly alleviate soil compaction and adhesion, promote straw decomposition, and enhance soil fertility (Yan et al., 2024). Mixed rotary tillage effectively increases soil organic matter (SOM) content, bacterial abundance, and diversity (Dong et al., 2025). In recent years, extensive research has demonstrated that no-till mulching significantly improves soil organic matter, promotes plant development, and increases crop yields (Li et al., 2024; Yang et al., 2024). Biologically enhanced techniques like field rapid composting and bioreactors accelerate straw decomposition into organic fertilizers by adding exogenous microbial inoculants (Liang et al., 2015; Rui et al., 2009). In karst regions, straw return practices are shifting from “simple incorporation” to “precision regulation” to address key constraints such as nutrient loss and soil fragility. However, current research on rice straw return in karst paddy fields still faces several challenges: most studies focus on single indicators like soil organic matter or yield, with limited systematic investigations into changes in soil physicochemical properties, microbial community structure, and ecosystem functions under different return methods. There is also a lack of theoretical support for technological adaptability.

In karst paddy fields, soil microbial communities play a crucial role in promoting straw decomposition and nutrient cycling (Huang et al., 2018). Their community structure, diversity, symbiotic patterns, and assembly processes actively respond to agricultural management practices in this region (Zhang et al., 2019; Wang et al., 2021). Although studies have reported individual effects of straw return on soil fertility or microbial communities, most research remains at the descriptive level. An integrated mechanistic framework—analyzing different straw return methods, soil environmental factors, microbial communities, including diversity, assembly processes, and network structure, and ecosystem functions (nutrient cycling, crop yield) within a unified research system—remains lacking. Specifically, are microbial community assemblies dominated by deterministic or stochastic processes under different tillage practices? How do these practices reshape the complexity and stability of microbial interaction networks? And how do these changes in microbial properties ultimately translate into crop yields? Answering these questions is crucial for deepening our understanding of the ecological effects of straw incorporation.

To address these issues, this study integrates field experiments, 16S rRNA high-throughput sequencing, multidimensional statistics, and partial least squares structural equation modeling (PLS-SEM) to systematically reveal the interaction mechanisms within the soil–microbe–crop system in karst paddy fields under different straw return practices. We hypothesize that: (1) different straw return methods act as unique habitat filters, differentially selecting microbial communities and leading to divergent community assembly processes and network structures; (2) this microbial community reshaping serves as a critical bridge, linking straw return to rice yield, potentially surpassing the importance of total soil nutrients themselves. This study aims to fill the theoretical gap in straw–microbe–soil–crop interactions within fragile karst ecosystems, providing scientific support for the precise application of straw return techniques in karst paddy fields.

## 2 Materials and methods

### 2.1 Test site overview

In 2022, the trial was conducted at the experimental base in Tuanze Community, Tuanze Town (27°48′ N, 107°5′ E). The trial period spanned one rice growing season. During the trial period (May to September), the average temperature in Tuanze Town ranged from approximately 18.8 °C to 27 °C, with average precipitation between 46 mm and 81 mm. The region’s average elevation is approximately 1,008 meters, with annual sunshine hours totaling about 1,114.7 h and average annual humidity at 82%. The base’s strata primarily consist of soluble rocks (such as limestone and dolomite), which have undergone long-term dissolution by flowing water, forming the material foundation of karst topography. The test soil is paddy soil originating from karst terrain—retained-type paddy soil (soil nomenclature, continuous naming system). Before the experiment, the fundamental properties of the soil (at a depth of 0–20 centimeters) were as follows: pH 6.64, soil organic matter (SOM) of 27.25 g/kg, total nitrogen (TN) of 1.48 g/kg, alkali-hydrolyzable nitrogen (AN) of 128 mg/kg, available phosphorus (AP) of 24.9 mg/kg, and available potassium (AK) of 102 mg/kg.

### 2.2 Experimental design and management

The experiment employed a completely randomized block design (RCBD) comprising five straw incorporation treatments and one control (CK), with three replicates per treatment, totaling 18 experimental plots. Each plot measured 30 m^2^ with a 50-cm buffer zone between plots. All treatments applied 3,000 kg hm^−1^ of straw (dry weight, consistent with conventional karst paddy field application rates), implemented as follows: PD (plow-deep tillage return): rice straw buried to a plow depth (>30 cm); RTM (rotary tillage incorporation *fastq* n): rice straw incorporated into the topsoil layer (0–20 cm) via rotary tillage; FRC (field rapid composting): rice straw was spread uniformly across the field surface. A decomposer (*Bacillus subtilis*) and urea (10% of straw weight) were added to adjust the carbon-to-nitrogen ratio and accelerate decomposition, followed by shallow rototilling (<20 cm); NT (no-till mulching): rice straw is evenly spread over the soil surface as mulch without any tillage; BR (bioreactor): pre-decomposed rice straw is treated with microbial inoculants in a bioreactor system and then incorporated into the soil via rotary tillage (<20 cm); and CK (control): No straw application, conventional fertilization only.

All treatments, including CK, received equal amounts of conventional fertilizer: 300 kg hm^−1^ compound fertilizer (N: P₂O₅: K₂O = 15:15:15) as basal fertilizer and 150 kg hm^−1^ urea as top dressing. In May, the rice variety “Yuzhenxiang” was transplanted at a density of 126,000 plants per hectare (row spacing: 25 cm × 33 cm) and harvested in September. Field management followed standard local practices.

### 2.3 Soil and plant sampling

In this experiment, rice seedlings are transplanted in May, followed by harvesting in September. All other management practices followed standard procedures, with consistent tillage management across treatments. At harvest, rice yield was measured across the entire plot. Soil samples were collected using the five-point sampling method. All samples were collected in sterile plastic bags and immediately transported to the laboratory using dry ice and divided into at least two portions as required by the study. A portion was stored at −80 °C for high-throughput DNA sequencing analysis, while another portion was air-dried for physicochemical parameter testing and analysis.

### 2.4 Analysis of crop yields and soil physicochemical properties

Surveys and analytical testing in accordance with the national standard “Evaluation Standard for Farmland Quality Grades” (GB/T 33469) were carried out. Second, yield surveys using the on-field yield estimation methods for grain, oilseed, and vegetable crops were carried out.

Soil sample pH was measured using a pH meter (PHS-3C, Shanghai Leichi) at a soil–water ratio of 1:2.5 (w/v). Organic matter content was determined using the potassium dichromate oxidation method. This technique oxidizes organic matter in the soil and calculates organic matter content based on the residual oxidant. This classical and widely applied method follows the protocol described by Walkley and Black (1934). Alkaline-hydrolyzed nitrogen was measured using the alkaline diffusion method, employing apparatus such as diffusion dishes and burettes. Alkaline solutions convert ammonium nitrogen and partial organic nitrogen in soil into ammonia gas, which diffuses and is absorbed by boric acid. Standard acid titration is then performed to calculate the alkali-hydrolyzable nitrogen content. Total nitrogen determination employs the Kjeldahl method: soil samples are digested to convert organic nitrogen into ammonia, which is absorbed by boric acid and titrated to measure total nitrogen content. The soil available phosphorus content was determined using the sodium bicarbonate extraction–molybdenum antimony colorimetric method, with a spectrophotometer (model UV-1800) employed for measurement. Available potassium is measured by flame photometry using a flame photometer (Model M410, Sherwood Scientific, United States). This method relies on potassium ions in the sample emitting light at specific wavelengths when excited in the flame. Potassium content is determined by measuring the light intensity. Soil bulk density was measured using the ring knife method (with three replicates per sample, each ring-cut sample having a volume of 100 cm^3^).

Rice yield determination employs the field yield measurement method, selecting representative plots during crop maturity for multi-point sampling to measure yield per unit area.

### 2.5 Soil DNA extraction and high-throughput sequencing

#### 2.5.1 Sample DNA extraction

Total microbial community genomic DNA was extracted using the E.Z.N.A.^®^ soil DNA kit (Omega Bio-tek, Norcross, GA, United States). The quality of extracted genomic DNA was assessed via 1% agarose gel electrophoresis. DNA concentration and purity were determined using the NanoDrop2000 (Thermo Scientific, United States).

#### 2.5.2 PCR amplification and sequencing library preparation

Using the extracted DNA as a template, the V3–V4 variable region of the 16S rRNA gene with the barcode-containing upstream primer 338F (5′-ACTCCTACGGGAGGCAGCAG-3′) and downstream primer 806R (5′-GGACTACHVGGGTWTCTAAT-3′) (Liu et al., 2016) to amplify the V3-V4 variable region of the 16S rRNA gene was amplified. The PCR reaction mixture consisted of 4 μL 5 × FastPfu Buffer, 2 μL 2.5 mM dNTPs, 0.8 μL forward primer (5 μM), reverse primer (5 μM) 0.8 μL, FastPfu polymerase 0.4 μL, 0.2 μL BSA, and template DNA 10 ng, supplemented with dd H₂O to 20 μL. After all samples were added, PCR amplification was performed with the following program: pre-denature at 95 °C for 3 min. A total of 27 cycles (denature at 95 °C for 30 s, anneal at 55 °C for 30 s, extend at 72 °C for 45 s) were performed, followed by a 10-min extension at 72 °C, and, finally, stored at 4 °C (PCR instrument: ABI GeneAmp^®^ 9700). The PCR products were detected and recovered via 2% agarose gel electrophoresis. Further, the products were recovered using a DNA gel purification kit (PCR Clean-Up Kit, China Yuhua). The purified products were quantified using Qubit 4.0 (Thermo Fisher Scientific, United States).

Following the completion of the aforementioned PCR amplification, sequencing library preparation is required. The NEXTFLEX Rapid DNA-Seq Kit is used to construct libraries from purified PCR products. The process begins with adapter ligation, followed by magnetic bead-based selection to remove self-ligated adapter fragments. Library template enrichment is achieved through PCR amplification technology, and finally, PCR products are recovered using magnetic beads to obtain the final library. Sequencing is performed using the Illumina NextSeq 2000 platform (Shanghai Meiji Biotechnology Co., Ltd.).

#### 2.5.3 High-throughput sequencing data analysis

Quality control of the raw sequencing reads from both ends was performed using the *fastq* (Chen S. et al., 2018) software (https://github.com/OpenGene/fastp, version 0.19.6). Perform assembly using *FLASH* (Magoč and Salzberg, 2011) (http://www.cbcb.umd.edu/software/flash, version 1.2.11): (1) Filtered bases with tailing quality scores below 20, set a 50 bp window, and if the average quality within the window fell below 20, truncated the trailing bases starting from the window. Filtered reads shorter than 50 bp after quality control and removed reads containing N bases; (2) Based on the overlap relationship between PE reads, merged paired reads into a single sequence with a minimum overlap length of 10 bp; (3) The maximum allowed mismatch ratio in the overlap region of assembled sequences is 0.2; sequences failing this criterion are filtered out; (4) Samples are distinguished based on barcodes and primers at the sequence ends, and sequence orientation is adjusted. Zero mismatches are allowed in barcodes, with a maximum of 2 mismatches in primers. Using *UPARSE v7.1* (Edgar, 2013) software,^1^ perform operational taxonomic unit (OTU) clustering on quality-controlled assembled sequences at 97% similarity and remove chimeras. Remove sequences annotated as chloroplast and mitochondrial genomes from all samples. Additionally, to minimize the impact of sequencing depth on subsequent α*-* and β-diversity analyses, all samples underwent sequence downsampling to 20,000 sequences (recommended practice). After downsampling, the average sequence coverage (Good’s coverage) per sample remained at 99.09%. OTU taxonomic annotation was performed using the RDP classifier (Wang et al., 2007) (http://rdp.cme.msu.edu/, version 2.11) against the Silva 16S rRNA gene database (v138) with a confidence threshold of 70%. Community composition at different taxonomic levels was then quantified for each sample. Functional prediction analysis of 16S rRNA genes was performed using *PICRUSt2* software (Douglas et al., 2020) (version 2.2.0).

### 2.6 Statistical analysis

The analysis conducted on the Meiji Bio Cloud Platform^2^ is as follows: alpha diversity metrics, including Chao1 and Shannon indices were calculated using *mothur* software (Schloss et al., 2009).^3^ Inter-group differences in alpha diversity were analyzed via the Wilcoxon rank-sum test. LEfSe analysis (linear discriminant analysis effect size) (Segata et al., 2011)^4^ (LDA >2, *p* < 0.05) to identify bacterial taxa with significant intergroup differences in abundance at the phylum and genus levels. Non-metric multidimensional scaling (NMDS) based on Bray–Curtis distances was performed to reveal intergroup differences in bacterial β-diversity. Distance-based redundancy analysis (db-RDA) was employed to investigate the influence of soil physicochemical parameters on bacterial community structure. Linear regression analysis was used to evaluate the impact of key soil physicochemical parameters identified by db-RDA on microbial alpha diversity indices. Species were selected for correlation network analysis based on Spearman correlations with |*r*| > 0.6 and *p* < 0.05 (Barberán et al., 2012).

This study employed Excel 2019 (Microsoft, United States) and SPSS 27 for data analysis, utilizing one-way analysis of variance (ANOVA) and Duncan’s multiple range test for multiple comparisons, while Pearson’s correlation coefficient was applied to assess correlations. Graphical representations were created using Graph 0.9.2 and Origin 2024 (OriginLab, OriginPro 2024, United States). PLS-PM analysis was performed using the “inner plot” function from the “plspm” package in R 4.5.1.

## 3 Results

### 3.1 Soil physicochemical properties and rice yield

**Figure 1:**
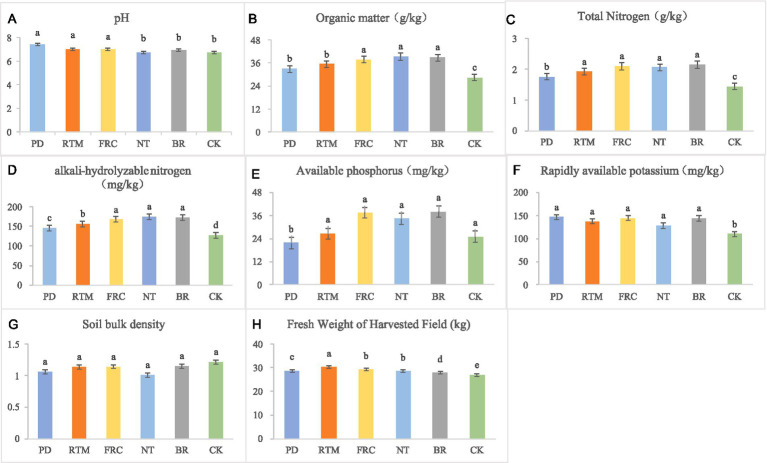
Soil pH **(A)**, SOM **(B)**, TN **(C)**, AN **(D)**, AP **(E)**, AK **(F)**, BD **(G)**, and yield **(H)** under different straw application methods. PD, plow-deep tillage straw incorporation; RTM, rotary tillage straw incorporation with mixing; FRC, field rapid composting of straw; NT, no-till straw mulching; BR, straw bioreactor; CK, blank control; SOM, soil organic matter; TN, total nitrogen; AN, alkali-hydrolyzable nitrogen; AP, available phosphorus; AK, available potassium; BD, soil bulk density.

Figure 1 compares the effects of different straw incorporation methods on soil physicochemical properties. Field rapid composting (FRC), no-till mulching (NT), and bioreactor (BR) significantly increased soil organic matter content. Straw deep plowing (PD) showed significantly lower available phosphorus than other treatments. This is likely attributed to soil pH exceeding 7 (PD: 7.4) following plow-based deep incorporation. While this pH level favors ammonium nitrogen retention, it also promotes calcium ion (Ca^2+^) binding with phosphate ions to form insoluble calcium phosphate (Ca₃(PO₄)₂), resulting in significantly reduced available phosphorus (<30 mg/kg). Except for the blank control (CK), all straw return methods significantly increased available potassium, with rapid decomposition (FRC) and no-till mulching (NT) yielding the best results. The fresh weight of rice harvested after straw incorporation via rotary tillage was significantly higher than other treatments (30.37 kg/plot). Compared to CK, all five straw application methods significantly increased rice fresh weight at harvest. Specifically, straw incorporation via rotary tillage increased fresh weight by 13.2%, while PD, FRC, NT, and BR increased it by 6.3, 9.3, 6.3, and 3.6%, respectively.

### 3.2 Effects of different straw application methods on soil microbial abundance and diversity

Analysis of bacterial communities from 18 samples yielded 750,960 valid bacterial gene sequences. Taxonomic annotation revealed that soil bacteria following different straw application treatments comprised 58 phyla, 183 classes, 430 orders, 677 families, and 1,269 genera.

**Figure 2:**
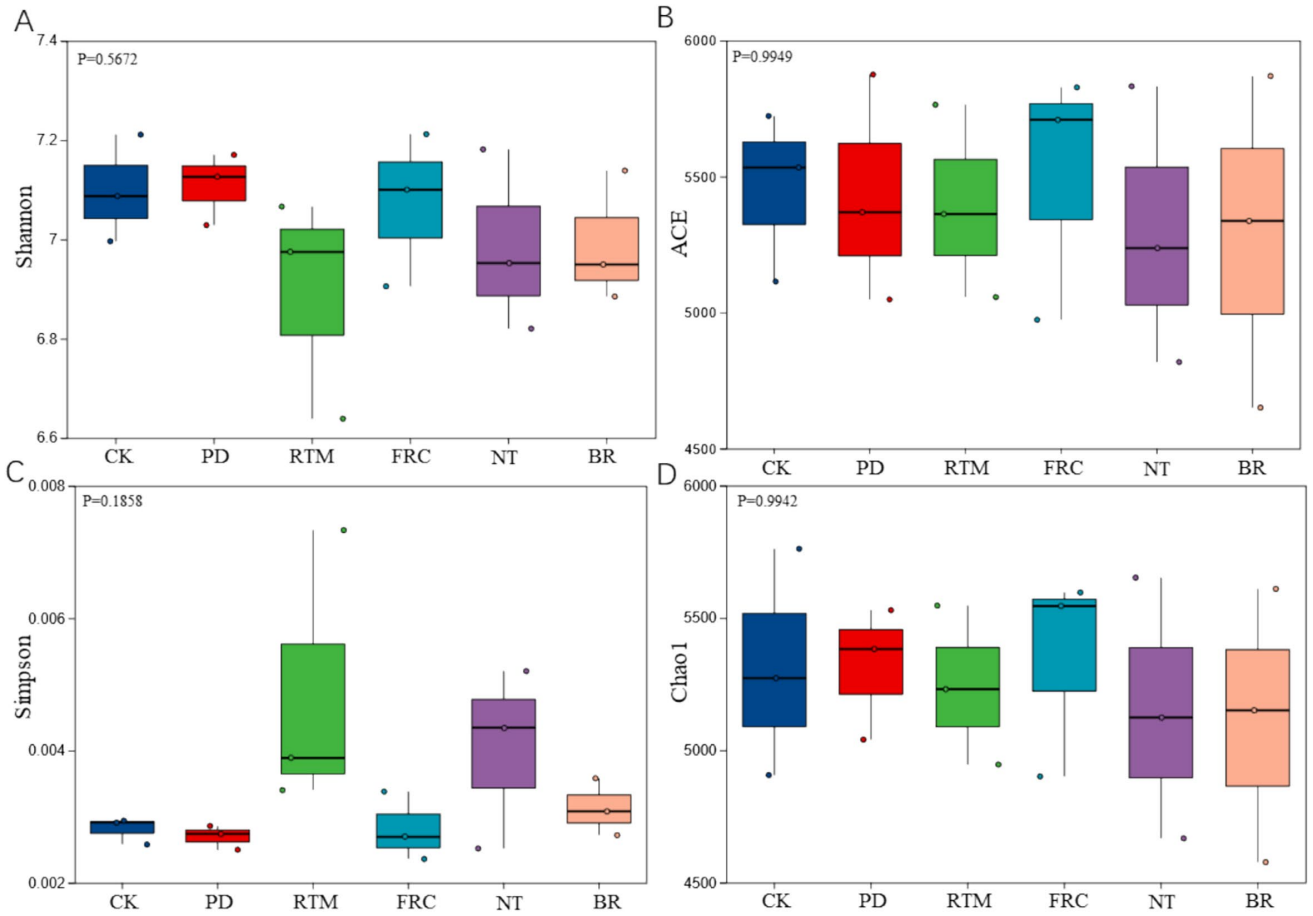
Shannon **(A)**, ACE **(B)**, Simpson **(C)**, and Chao1 **(D)** indices of soil microbial communities under different straw application methods. Intergroup differences were analyzed using the Wilcoxon rank-sum test (*p* > 0.05 indicates no significant difference). PD, plow-deep tillage and straw incorporation; RTM, rotary tillage and mixed incorporation of straw; FRC, field rapid composting of straw; NT, no-till mulching and straw incorporation; BR, straw bioreactor; CK, blank control.

In this study, microbial community α-diversity was assessed using multiple diversity indices. Results indicated no significant differences in α-diversity among different straw application methods (see Figure 2).

**Figure 3:**
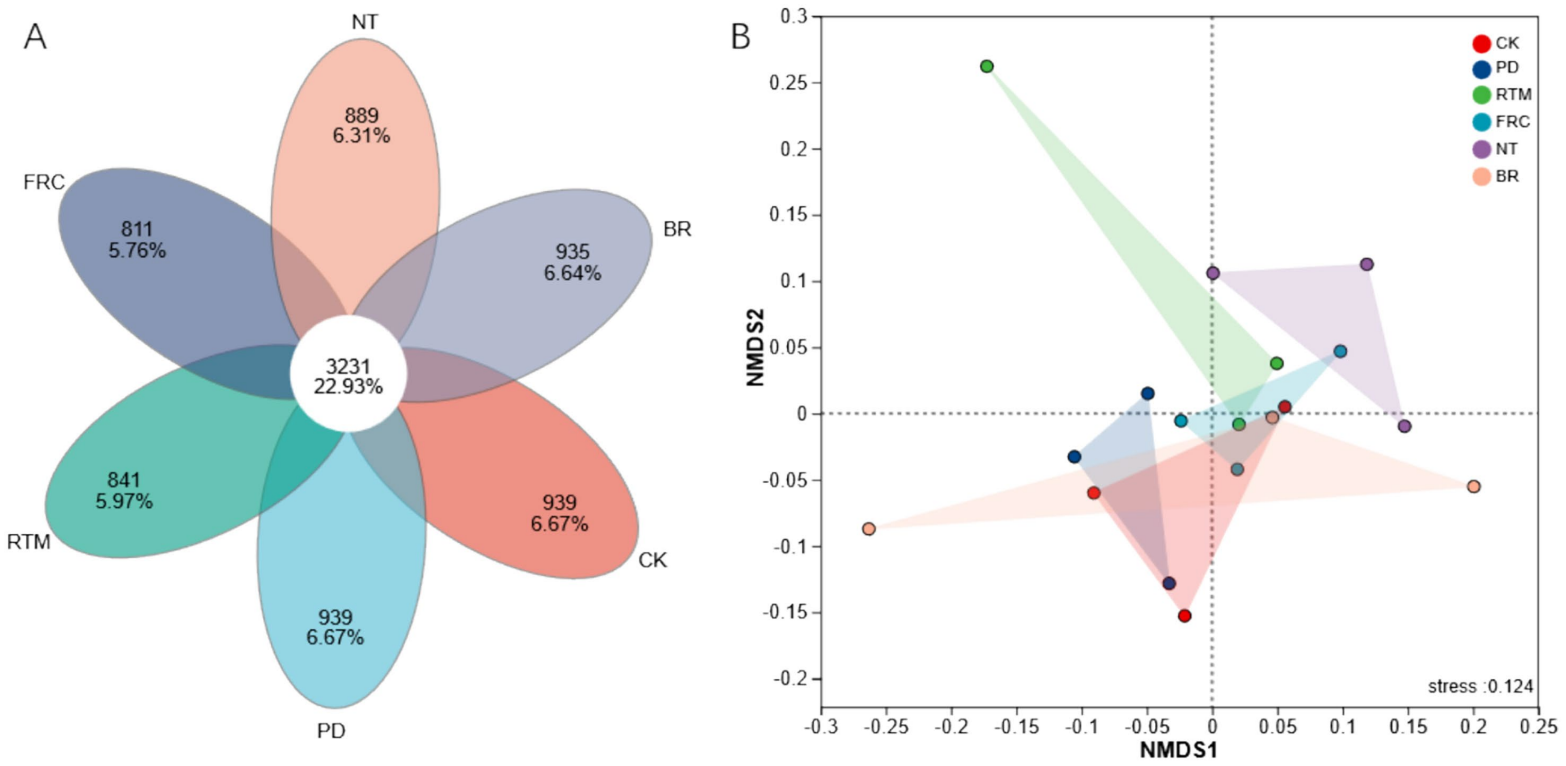
Differences in the number of unique and shared OTUs (operational taxonomic units) in soil microorganisms (Venn diagram) **(A)**. Non-metric multidimensional scaling (NMDS) of soil bacterial communities under different straw application methods **(B)**. PD, plow-incorporated deep tillage of straw; RTM, rotary tillage with mixed incorporation of straw; FRC, field rapid composting of straw; NT, no-till mulching of straw; BR, straw bioreactor; CK, CK, blank control.

Figure 3A shows the Venn diagram illustrating the number of shared and unique microbial species among different straw treatment methods. The central region, representing 3,231 microbial species (22.93%), indicates species common to all treatments, while each treatment also possesses its own unique microbial species. To further investigate the similarities and differences in microbial community structure across different treatments, non-metric multidimensional scaling (NMDS) was employed for additional analysis at the ASV (amplicon sequence variant) level. Figure 3B shows that sample points from certain treatments cluster together, indicating relatively similar microbial community structures under these treatments. The stress value of 0.124 indicates a good fit of data points in the two-dimensional space, suggesting reliable results.

### 3.3 Straw application methods drive bacterial community composition and function

#### 3.3.1 The composition of microbial communities varies depending on the method of straw application

**Figure 4:**
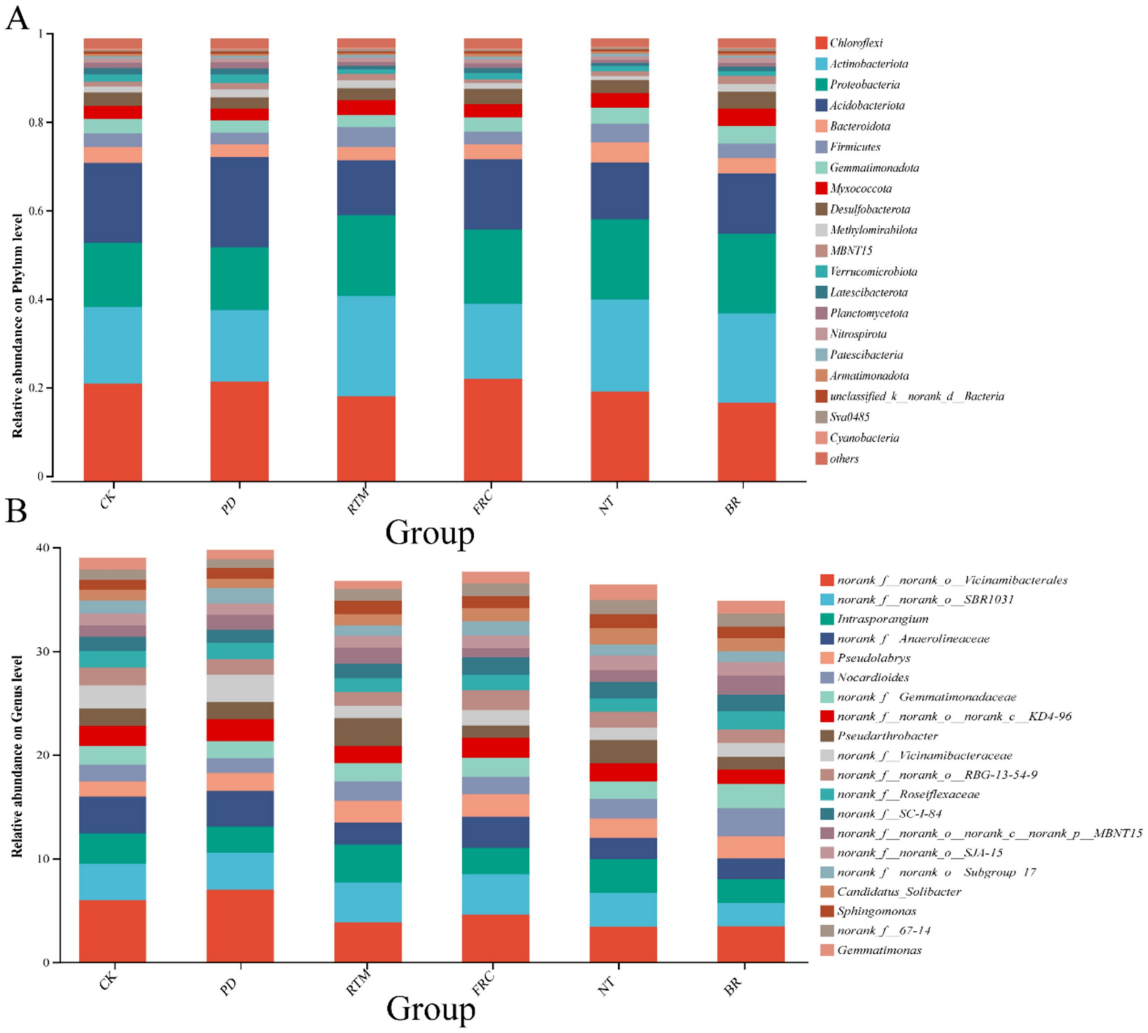
Effects of different straw application methods on dominant microorganisms at the genus level **(A)** and species level **(B)**. PD, plow-incorporated straw deep tillage; RTM, rotary tillage mixed-incorporation straw; FRC, field rapid composting straw; NT, no-till straw mulching; BR, straw bioreactor; CK, blank control.

As shown in Figure 4, the phyla Proteobacteria, Bacteroidetes, Actinobacteria, Chloroflexi, Planctomycetes, Gemmatimonadetes, Nitrospirae, Verrucomicrobia, Acidobacteria, and Firmicutes are the primary microbial groups involved in various ecological functions, such as material cycling and energy metabolism. At the genus level, *Comamonadaceae*, *Sphingomonadales*, *Sphingomonadaceae*, *Burkholderiales*, *Sphingobacteriaceae*, *Sphingobacteriales*, *Comamonadaceae*, *Sphingomonadales*, *Sphingomonadaceae*, and *Burkholderiales* occupy significant positions within microbial communities.

#### 3.3.2 The function of microbial communities varies depending on the method of straw application

**Figure 5:**
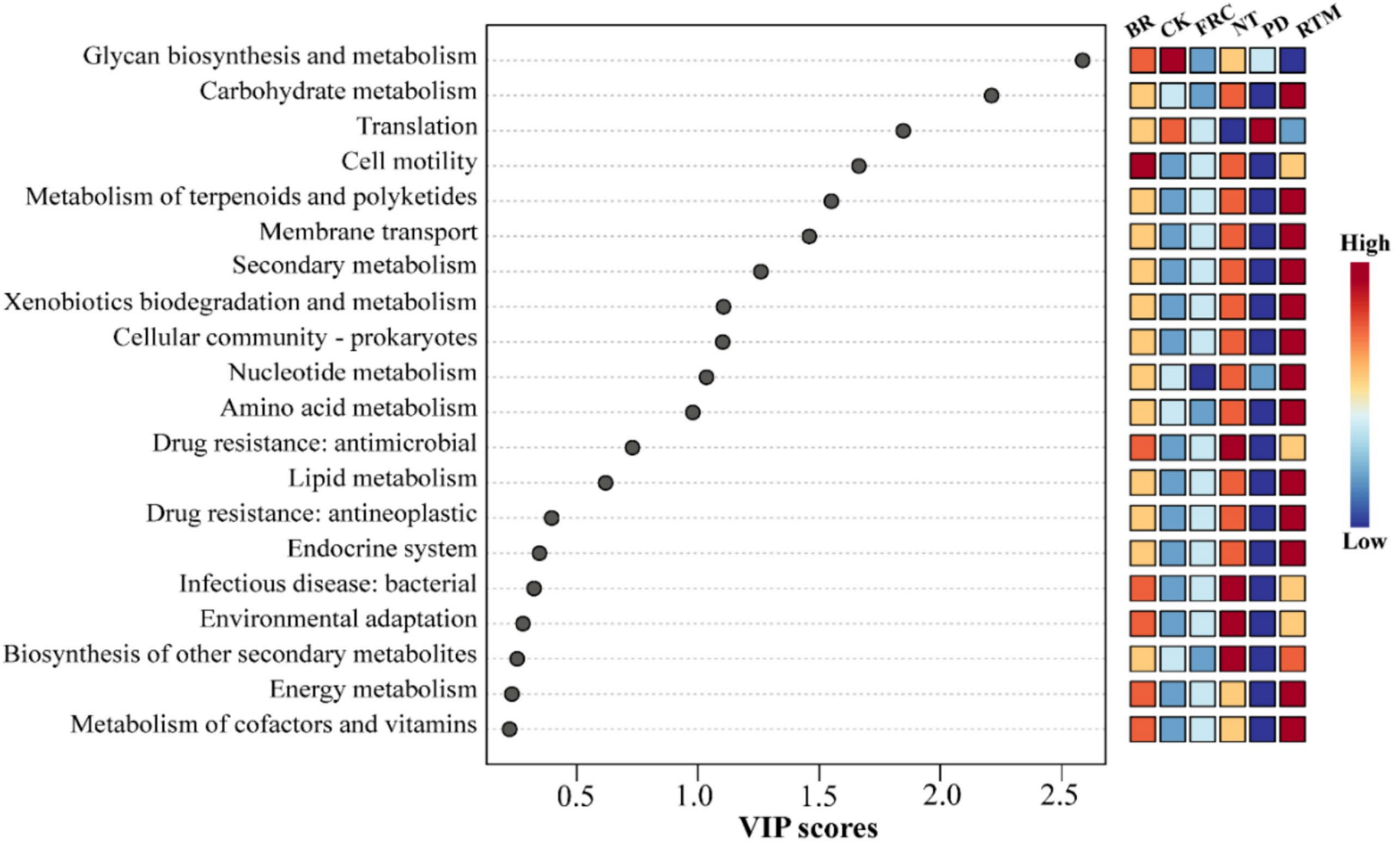
Predicted distribution of soil microbial functions after different straw application methods: PD, plow-incorporated straw deep tillage; RTM, rotary tillage mixed-incorporation straw; FRC, field rapid composting straw; NT, no-till mulching straw; BR, straw bioreactor; CK, blank control.

Based on the functional prediction results of *16S rRNA* gene sequences using PICRUSt2 software, combined with variable importance projection (VIP) scores and grouped heatmaps, the differential response patterns of soil microbial functional pathways under control (CK) and five straw returning treatments were systematically evaluated (Figure 5). The results showed that the VIP values of carbon metabolism related pathways (glycosylation biosynthesis and carbohydrate metabolism) ranked high (VIP >1.0), and the pathway activity of the straw treatment group (especially RTM and FRC) was significantly higher than that of the CK (*p* < 0.05), manifested as a red high expression cluster in the heatmap; the basal metabolism and synthesis pathways, namely translation related pathways, maintained high activity in all treatment groups, but the activity of the straw treatment group (especially BR and NT) was slightly higher than that of the CK (*p* > 0.05); The specific functional pathway (degradation and metabolism of foreign compounds) showed significantly high activity in BR and FRC treatments (VIP >1.2, *p* < 0.01), while its activity was lower in PD and NT treatments.

### 3.4 Soil bacterial assembly processes and network analysis under different straw application methods

**Figure 6:**
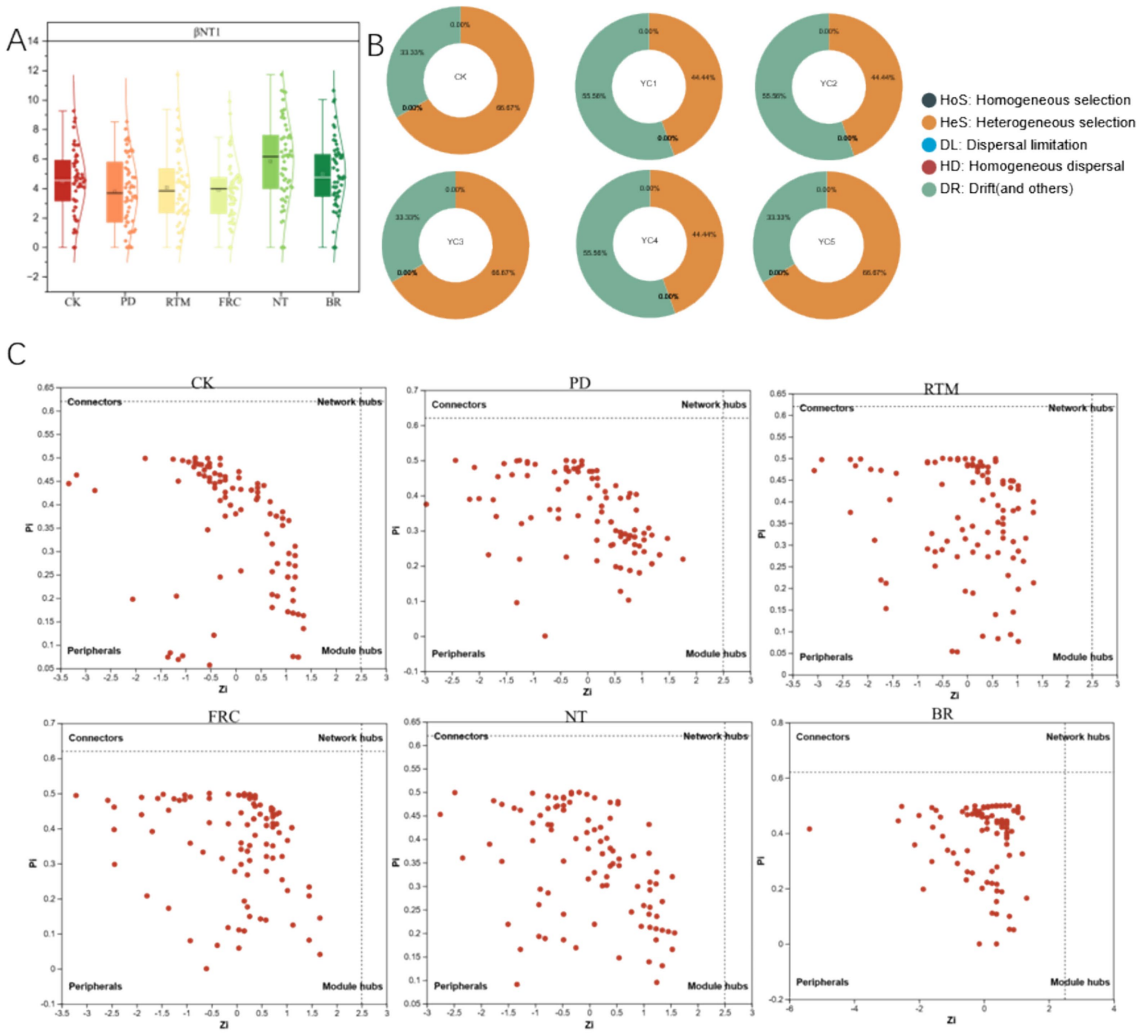
Effects of different straw return methods on bacterial community assembly. **(A)** βNTI values (|βNTI| >2 indicates deterministic processes dominate, |βNTI| <2 indicates stochastic processes dominate). **(B)** Proportion of community assembly processes (HoS, homogenous selection; HeS, heterogeneous selection; DL, diffusion limitation; HD, homogeneous diffusion; DR, ecological drift). **(C)** Network topology (constructed based on Spearman correlation |*r*| > 0.6, *p* < 0.05; node colors represent different phylum-level taxa). PD, straw plow-tillage deep incorporation; RTM, straw rotary tillage mixed incorporation; FRC, straw field rapid decomposition; NT, straw no-till mulching; BR, straw bioreactor; CK, blank control.

As shown in Figure 6A, compared with the control (CK), the β-nearest taxon index (βNTI) values for different straw application methods were significantly higher than CK, indicating that these application methods increased the diversity of community structure. Among them, NT and BR had the highest βNTI values, suggesting that these two application methods had the greatest impact on community structure and could lead to significant changes in community structure. Additionally, the NT and BR treatments exhibited relatively higher proportions of homogenous selection (HoS) (Figure 6B), suggesting these application methods tend to promote homogenous selection within the community. We also observed that the network topologies of all five treatments were more dispersed compared to CK (Figure 6C), with increased node distribution in the connectors and peripherals regions. This suggests that straw application methods disrupted the original network structure, increasing network complexity and instability. The NT and BR treatments exhibited the most dispersed network topologies, with the highest node distribution in the peripherals region, indicating that these two application methods caused the strongest disruption to the network structure.

**Figure 7:**
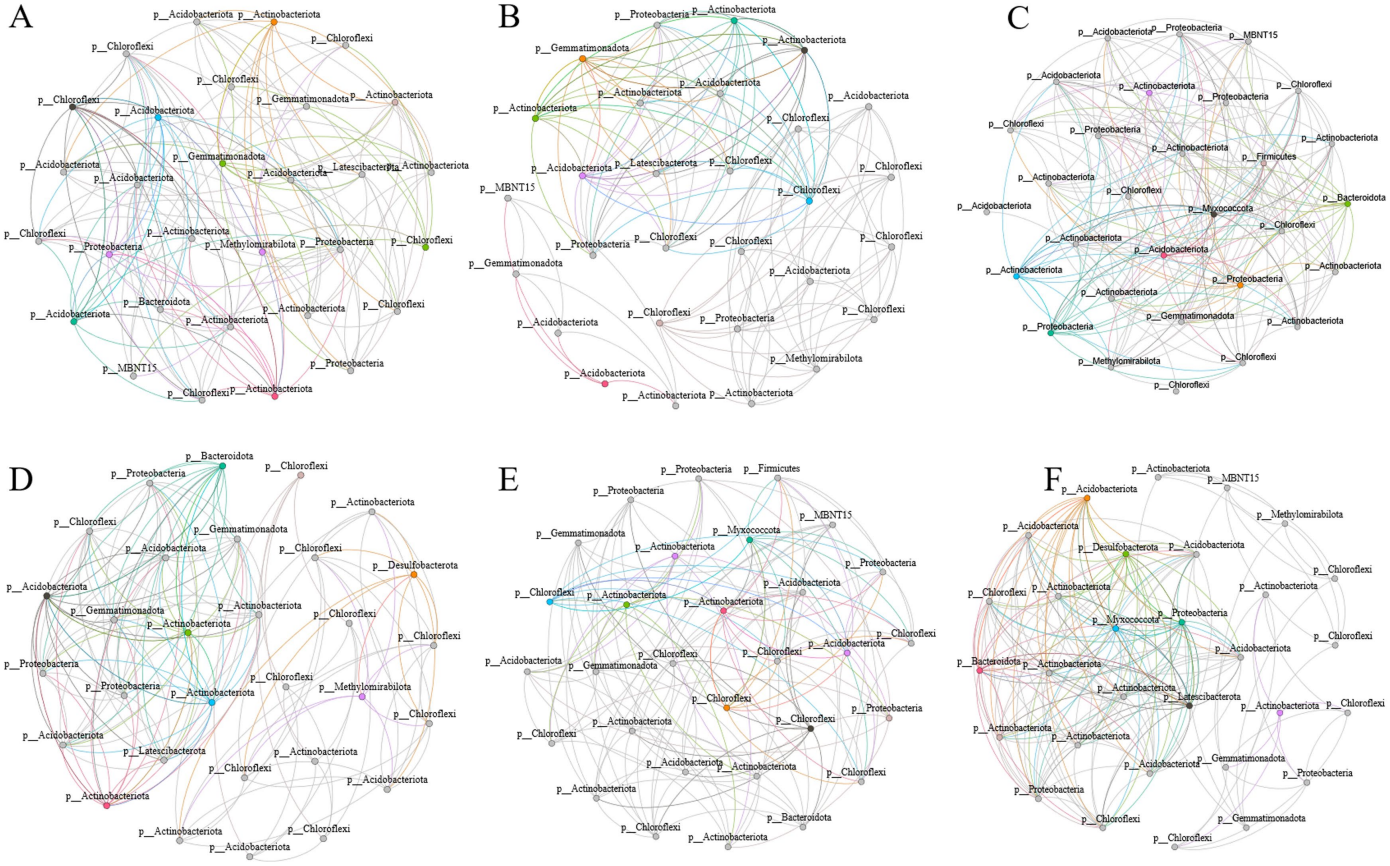
Network structure analysis of microbial communities under different straw treatments. **(A–F)** Correspond to the following treatments in sequence: CK, control treatment; PD, straw plowing and deep tillage for incorporation; RTM, rotary tillage with mixed incorporation of straw; FRC, rapid field composting of straw; NT, no-till straw mulching; BR, straw bioreactor.

Analysis of microbial community network structures under different straw treatments revealed that the BR treatment exhibited the densest network connectivity (Figure 7), with the highest average degree of 12.200 and the highest proportion of positive correlations at 51%. These results indicate that the microbial network structure under this treatment was the most tightly knit, featuring the greatest number of positive microbial interactions. Additionally, we observed that the NT treatment demonstrated the highest modularity, suggesting that the community structure under this treatment was the most pronounced (see Table 1).

**Table 1:** Analysis of network structural indices of microbial communities under different straw treatments.

Network metrics	CK	PD	RTM	FRC	NT	BR
Number of nodes	30	29	30	30	30	30
Number of edges	148	143	184	156	136	183
Average degree	9.867	9.862	12.267	10.400	9.067	12.200
Modularity	0.574	0.550	0.495	0.484	0.657	0.288
Number of communities	3	3	3	3	3	3
Network diameter	1	1	1	1	1	1
Network density	0.340	0.352	0.423	0.359	0.313	0.421
Average clustering coefficient	1	1	1	1	1	1
Positive correlation (%)	48	47	49	47	49	51
Negative correlation (%)	52	53	51	53	51	49

### 3.5 The impact of environmental factors on microbial communities

**Figure 8:**
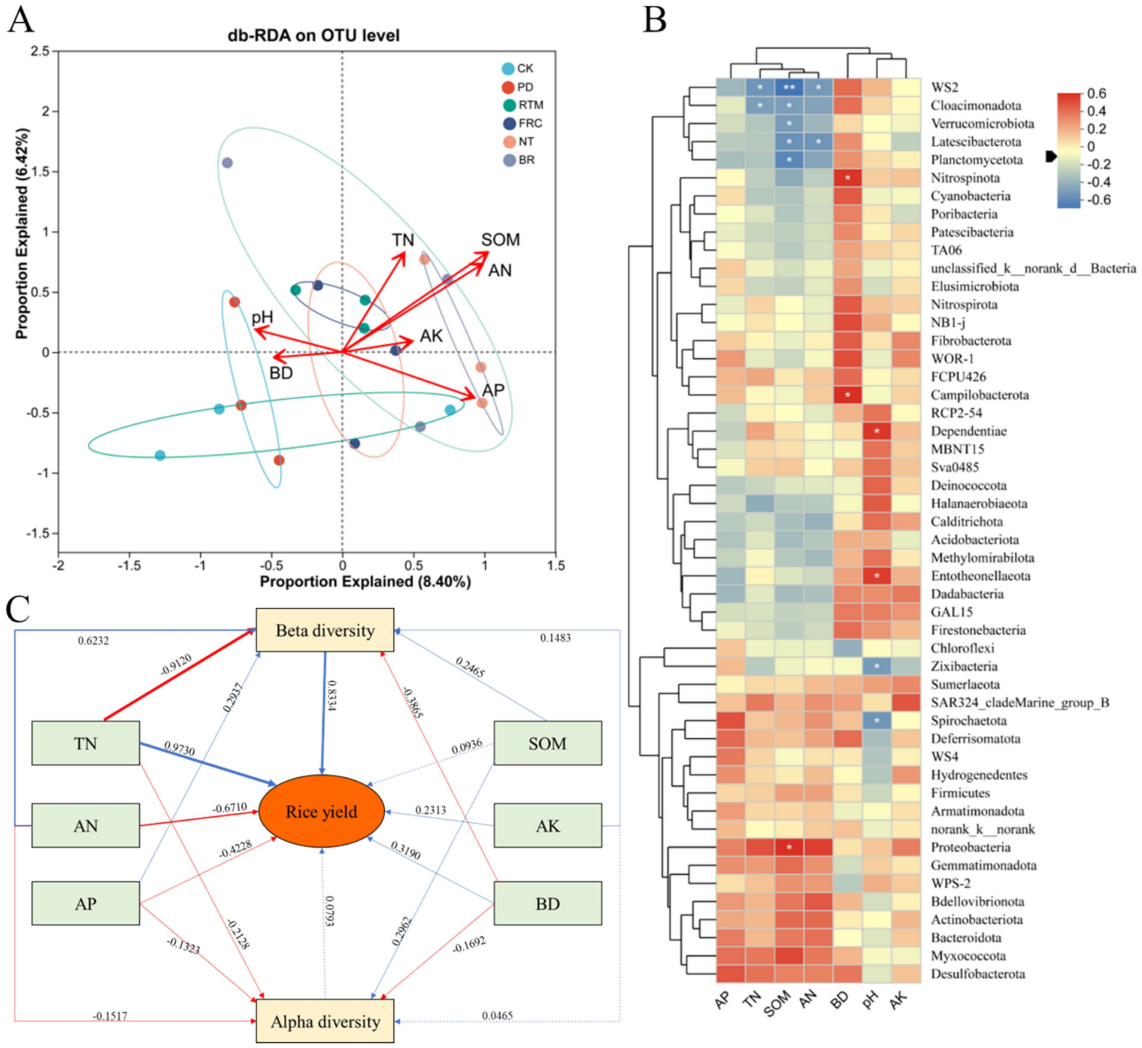
Redundancy analysis (RDA) of environmental factors associated with microbial communities **(A)**. Heatmap showing correlations between species relative abundance and family-level environmental factors **(B)**. PLS-SEM path analysis model **(C)**. Red arrows indicate negative effects (negative correlations). Blue arrows indicate positive effects (positive correlations). Line thickness indicates correlation strength, with thicker lines representing stronger correlations.

The db-RDA analysis (distance-based redundancy analysis) revealed that environmental factors such as pH, BD, AK, AP, SOM, and AN significantly influenced microbial community structure and relative abundance (Figure 8A), acting as key drivers of microbial community variation. Additionally, we observed that the BR sample point exhibited a distinct location, indicating its microbial community effects differed substantially from other treatments. For instance, FRC (straw field rapid decomposition) may influence microbial communities by altering factors such as SOM and AN. Correlation heatmaps similarly revealed treatment-specific effects on microbial communities (Figure 8B). Under PD and RTM treatments, certain Actinobacteria phyla exhibited elevated abundances. Treatment impacts on environmental factors also varied: PD and RTM primarily influenced TN and AN, while NT and BR significantly affected SOM and AN. Interestingly, BD exhibited distinct characteristics among these treatments. By significantly altering pH and AK environmental factors, BD treatment exerted substantial effects on microorganisms belonging to the Chloroflexi and Proteobacteria phyla. This suggests that BD treatment may selectively promote or inhibit the growth of specific microorganisms by regulating soil pH and available potassium content.

PLS-SEM analysis (goodness-of-fit = 0.54; Figure 8C) indicates that TN (path coefficient = 0.97, *p* < 0.001) and β-diversity (path coefficient = 0.83, *p* < 0.001) exert strong positive effects on rice yield, suggesting that changes in TN and biodiversity significantly influence rice production. Furthermore, TN exerts a significant negative effect on β-diversity (path coefficient = −0.91, *p* < 0.001). AN (path coefficient = −0.67, *p* < 0.001) and AP (path coefficient = −0.42, *p* < 0.001) exerted significant negative effects on rice yield, yet exerted positive effects on β-diversity (path coefficient = 0.62, *p* < 0.001) and α-diversity (path coefficient = 0.29, *p* < 0.001). α-diversity (path coefficient = 0.08, *p* < 0.001) exerted a slight positive effect on rice yield.

## 4 Discussion

Our research extends beyond conventional reports on the effects of straw on soil fertility and crop yields. By focusing on the unique geological context of karst paddy fields, we have uncovered the microbial mechanisms underlying straw return practices. The distinctiveness of the karst geological setting significantly modulates the effects of different straw return methods. We found that different straw treatment methods can act as powerful habitat filters, exerting directional drives on bacterial community assembly, network structure, and specific functions. Crucially, it is not isolated soil chemical properties that determine final agroecosystem outcomes, but rather their interplay with microbial responses. This effectively explains the disconnect between the treatment with the highest soil SOM accumulation (NT) and the highest grain yield (RTM).

### 4.1 Differential regulation of soil fertility and crop yield by straw returning: the “synchronization effect” of microorganisms

Consistent with most studies (Bagheri Novair et al., 2024; Zhou et al., 2017; Zhao et al., 2017), all straw return treatments significantly increased soil organic carbon and nutrient pools compared to the control. However, the unique conditions of karst paddy fields accentuated the differences in mechanisms and efficacy among various straw return methods. In non-karst regions with thick soil layers, such as the Loess Plateau, deep plowing helps break the plowpan and enhance fertility (Zhang et al., 2022). However, in karst areas with thin soil layers, deep plowing exacerbates soil erosion, reduces available phosphorus content (Figure 1E), and significantly diminishes yield gains. Studies in non-karst paddy fields on the East China Plain indicate that rapid in-field decomposition treatment offers distinct yield advantages (Han et al., 2022). However, in our research, the high calcium content in karst soils causes nutrients to readily bind and immobilize with calcium during straw decomposition (Meng et al., 2023), resulting in smaller yield gains than RTM. This highlights the selective influence of karst soil characteristics on return methods. Under NT treatment, SOM accumulation was significantly higher than in other treatments, increasing by 38.16% compared to CK (Figure 1). This aligns with Liu et al.’s findings, where surface cover reduces soil disturbance, thereby minimizing aggregate disruption and lowering microbial organic matter mineralization rates (Wen et al., 2023; Liu et al., 2023). For fragile karst thin soils, this approach maximizes the reduction of nutrient and soil loss. The contrast between NT (high SOM) and RTM (high yield) indicates that crop yield is an emergent property of multiple interacting factors, rather than a simple function of total nutrient pools. We hypothesize that lower soil temperatures under NT treatment delayed crop germination (Chen G. et al., 2018), and the slow nutrient release dominated by phyla like Chloroflexi (Figure 4A) failed to match the rice nutrient demand peak (Mengistu et al., 2024), ultimately limiting its yield potential. Conversely, RTM treatment enriched bacterial genera with nitrogen-fixing capabilities, such as *Pseudarthrobacter*, *Nocardioides*, and *Pseudolabrys*, alongside *Sphingomonas*—a genus exhibiting nitrogen fixation, phosphorus solubilization, and plant growth promotion (Gatheru Waigi et al., 2017) (Figure 4B). This enrichment of functional microbial communities directly or indirectly enhances nutrient utilization, thereby promoting rice growth. Concurrently, PICRUSt2 prediction results indicate that carbon metabolism-related pathways (glycosylation biosynthesis, carbohydrate metabolism) exhibit the highest activity in RTM-treated samples (VIP >1.0, *p* < 0.05) (Figure 5). This indicates that the microbial community under this treatment rapidly decomposes straw to release readily available nutrients, precisely meeting the urgent need for “rapid nutrient supply” in low-fertility karst soils, ultimately achieving the highest yield. This highlights a critical insight: management strategies should not solely aim to maximize soil nutrient reserves. Optimizing nutrient release timing and crop uptake dynamics is equally vital, with microbial communities playing a pivotal role as “dispatchers” in this process (Cai et al., 2024).

### 4.2 Regulation of bacterial communities by straw returning to fields: β-diversity is key

In our study, no significant changes in α-diversity indices were observed among different treatments. This may be attributed to the adaptation of soil microbial communities to the unique low-fertility environment in long-term karst paddy field practices. Short-term straw application does not disrupt the balance of native soil species diversity. Similarly, in non-karst arid areas with purple soils, α-diversity showed no significant changes after straw incorporation. This aligns with our findings (Beschoren da Costa et al., 2022). However, β-diversity analysis revealed significant segregation in soil bacterial community structures among treatments via NMDS ordination (Figure 3B). These changes indicate that the core impact of straw application on soil lies not in species abundance but in altering species composition and relative abundance (Beschoren da Costa et al., 2022). Specifically, PD and RTM treatments altered soil physical conditions (e.g., aeration, straw distribution) through tillage, selecting aerobic groups like Proteobacteria; FRC and BR directly introduced new microbial functional groups via exogenous inoculation of microbial agents. The NT treatment maintained topsoil stability but increased microenvironmental heterogeneity, enriching degradative groups like Chloroflexi and Actinobacteriota (Zhang et al., 2021; Edwards et al., 2015; Lu et al., 2006). These interventions act as environmental filters, selectively determining which groups adapt to the specific new conditions they create (e.g., aerobic decomposers in tilled systems and facultative anaerobes under cover), leading to a restructuring of soil community composition toward adaptive configurations (Beschoren da Costa et al., 2022; Wang et al., 2024).

### 4.3 Microbial co-occurrence networks and assembly processes reveal mechanisms of community organization

To gain deeper insights into the mechanisms organizing bacterial communities under different straw management practices, we employed a null model analysis. βNTI analysis revealed that 65% of all straw return treatments exhibited |βNTI| >2 (Figure 6A), indicating that deterministic processes—particularly heterogeneous selection—were the dominant drivers of community differentiation across straw treatments (Cui S. et al., 2025). This contrasts with the prevailing conclusion for non-karst soils that “deterministic and stochastic processes act synergistically” (Liang et al., 2025)—the core reason being that karst soils amplify the screening pressure exerted by soil environmental changes (e.g., pH, nutrient availability) induced by straw return on microorganisms, resulting in a more pronounced “habitat filter” effect, thus validating our Hypothesis 1. Heterogeneous selection was more prevalent in NT and BR treatments. This is likely because NT treatment reduced water evaporation through surface soil cover, maintaining relatively stable soil moisture conditions and a low-oxygen environment, thereby selecting for adapted facultative anaerobic microbial communities (Giller et al., 2015; Taskin et al., 2021). Simultaneously, the high SOM content in this treatment intensified homogenous selection for carbon-fixing groups, promoting carbon-fixing community assembly. This facilitates organic matter accumulation but results in slower nutrient release rates due to soil microenvironmental influences. The BR treatment likely synergistically combines exogenous microbial inoculation with straw pre-decomposition to create high nutrient availability (total nitrogen, alkali-hydrolyzable nitrogen) and a stable soil microenvironment. This promotes enrichment of nutrient cycling microbial communities, achieving enhanced fertilizer efficiency.

Co-occurrence network analysis revealed that the BR treatment exhibited the highest connectivity and lowest modularity, indicating closer collaborative relationships among microorganisms under this treatment. Correspondingly, key groups in this treatment included the family Monodionaceae (Phylum Proteobacteria) and the families Sphingomonas and Rhizobium (Phylum Bacteroidetes). These three groups formed an ecological functional network of “decomposition-transformation-retention” in the soil environment through complementary functions (Seitz et al., 2022; Nagar et al., 2020; Steenhoudt and Vanderleyden, 2000). The presence of these key groups further enhanced the complexity and stability of the soil microbial network. In contrast, the NT network exhibited the highest modularity, reflecting significant ecological niche differentiation within its community structure—different modules corresponded to specific microenvironments such as straw surfaces and soil particles, as well as functional niches like lignin decomposition and nitrogen mineralization. This differentiation enhances functional redundancy, thereby strengthening the stability of carbon sequestration processes. When compared to non-karst soil networks, which typically exhibit more balanced structures with moderate modularity and connectivity, this further highlights the unique regulatory role of karst environments in shaping microbial interaction patterns.

### 4.4 An integrated framework: the interconnectedness of soil properties, microbiomes, and yields

Integrating the unique characteristics of karst paddy fields, we synthesized the complex relationship between environmental factors and microbial populations into a systematic mechanistic framework (Figure 8): Total nitrogen (TN) emerged as the most potent positive driver of yield, a finding particularly significant in karst soils where nitrogen is the primary nutrient limiting factor (Chen et al., 2019). The increase in TN from straw return effectively alleviated this constraint. Bacterial β-diversity emerged as the second-strongest direct positive driver with a path coefficient of 0.83 (supporting Hypothesis 2). This intriguing finding indicates that microbial community composition itself is a key determinant of yield, consistent with previous research. Specific community structures—such as those rich in plant-root-associated symbionts, nitrifying bacteria, and efficient decomposers—are crucial for enhancing soil productivity. The inverse effect of TN on β-diversity suggests a “eutrophication filtering” effect. In other words, soils with high nitrogen content may suppress certain oligotrophic or specialist bacteria, leading to greater functional similarity within bacterial communities. This highlights the potential unintended consequences of nitrogen enrichment. The model indicates minimal direct effects on α-diversity, reinforcing the concept that microbial functions—jointly determined by species identity and interactions—significantly influence agroecosystem processes far more than species richness alone (Moulton-Brown et al., 2022). Spearman correlation matrices reveal strong associations between bacterial phyla and soil properties. For instance, the Proteobacteria phylum showed strong positive correlations with TN, SOM, SAN, and AP. Certain members of Proteobacteria can secrete enzymes like urease and catalase that promote carbon and nitrogen cycling (Rampelotto et al., 2013; Cui Y. et al., 2025). Conversely, the Acidobacteria phylum exhibited negative correlations with most nutrients, consistent with their oligotrophic lifestyle (Barns et al., 1999), which is more common in karst soils with low fertility. Chloroflexi and Verrucomicrobiota showed negative correlations with multiple soil properties, potentially indicating adaptation to more stressed or nutrient-deficient conditions (Finn et al., 2020). These correlations further reinforce the specific mechanism in karst soils: “straw return → habitat alteration → community reshaping → functional enhancement → yield increase.”

**Figure 9:**
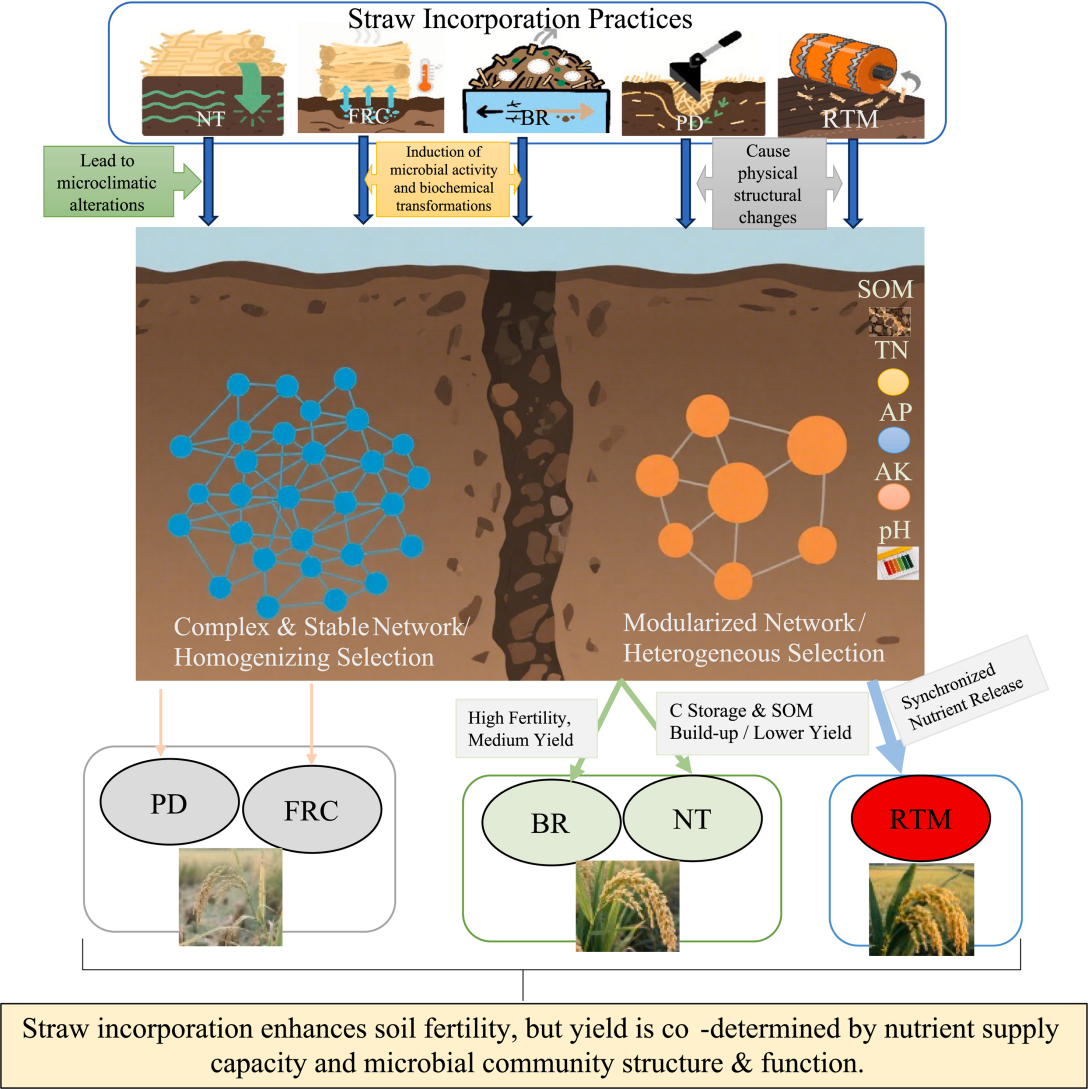
Insights into the mechanism by which straw returning to fields regulates soil fertility and crop yield through modulation of microbial communities.

In summary, we propose an integrated mechanism (Figure 9): Different straw return methods create distinct biogeochemical habitats → These created habitats act as habitat filters, imposing variable selection on microbial pools → This deterministically shapes distinct community structures (β-diversity) and network structures → Microbial configurations drive differential soil functions (e.g., carbon sequestration or nutrient transformation) → leading to variations in soil fertility and crop yields. Overall, RTM achieves optimal synergistic enhancement of soil nutrients and crop yields by assembling a balanced and interacting microbiome, aligning with karst regions’ “yield-first” requirements, while NT and BR demonstrate distinct advantages in soil conditioning and rapid nutrient cycling, respectively.

### 4.5 Research limitations

This study represents a short-term experiment conducted during a single growing season. The cumulative effects of long-term straw incorporation on bacterial community succession and soil fertility require further investigation and validation. Additionally, the analysis focused solely on bacterial communities, neglecting other microbial groups such as fungi and archaea. Future research should include long-term, fixed-site experiments spanning 5 years or more, combined with metagenomic sequencing to further elucidate the mechanisms of microbially mediated nutrient cycling.

## 5 Conclusion

This study, combining field experiments with *16S rRNA* sequencing, elucidated the mechanisms by which different straw return methods in karst paddy fields influence soil bacterial communities and rice yield: (1) all five straw return treatments enhanced soil fertility, with NT and BR showing the greatest effects on increasing soil organic matter and total nitrogen accumulation, respectively, while RTM demonstrated the highest yield increase. (2) Straw incorporation did not affect bacterial α-diversity but significantly altered β-diversity, community assembly processes, and network structure: deterministic processes dominated assembly; NT and BR enhanced homogenous selection; BR formed the most complex network; NT exhibited high modularity. (3) PLS-SEM models confirmed that soil total nitrogen and bacterial β-diversity are the most critical factors influencing rice yield. Based on research findings, balancing short-term benefits with long-term sustainability, RTM treatment requires no additional inputs, is compatible with conventional machinery, and involves low labor costs, making it suitable for large-scale agricultural cultivation in similar agroecosystems. While BR can rapidly improve soil fertility, it necessitates additional investments in microbial inoculants and decomposition costs, making it more appropriate for small-scale, high-value agricultural production.

## Data Availability

The raw reads of sequencing data is available at NCBI BioProject SRA database under the accession number PRJNA1370614.

## Footnotes

1 http://drive5.com/uparse/

2 https://cloud.majorbio.com

3 http://www.mothur.org/wiki/Calculators

4 http://huttenhower.sph.harvard.edu/LEfSe
